# Peasant perception of beekeeping constraints and practices in large honey production areas in Burkina Faso

**DOI:** 10.1186/s13002-024-00690-z

**Published:** 2024-07-17

**Authors:** Oswald Gilbert Dingtoumda, Marcellin Yamkoulga, Souhaïbou Sawadogo, Koï Wenceslas Kam, Zakaria Ilboudo

**Affiliations:** 1 Laboratoire d’Entomologie Fondamentale et Appliquée (LEFA), UFR, SVT, Université Joseph KI-ZERBO, 06 BP 9499, Ouagadougou, Burkina Faso; 2 Département Environnement et Foret (DEF), Institut de L’Environnement et de Recherches Agricoles (INERA), Station de Saria, BP 10, Koudougou, Burkina Faso; 3 Institut Supérieur du Développement Durable (ISDD), Université de Fada N’gourma, BP 54, Fada N’gourma, Burkina Faso

**Keywords:** Beekeeping, Agriculture, Phytosanitary products, Impact, Burkina Faso

## Abstract

**Background:**

In recent decades, agricultural landscapes have been profoundly modified due to the intensification of agriculture, therefore leading to significant disturbances in all components of biodiversity. A survey on the knowledge of beekeeping realities and the use of phytosanitary products in areas of high honey production in Burkina Faso was carried out. Beekeeping realities design the state of beekeeping activities in the study localities.

**Methods:**

The objective of this survey was to characterize Beekeeping operations and to assess the level of knowledge of beekeepers on the effects of the use of phytosanitary products through different beekeeping and agricultural practices. In this sense, 113 farmer beekeepers from the Boucle du Mouhoun, Hauts-Bassins and Nord regions in Burkina Faso were surveyed about their different beekeeping practices.

**Results:**

The results obtained indicated that beekeeping is a secondary activity (96.47%) and is mainly practiced by men (90.27%). The respondents have mostly an average of 22 traditional hives. The majority of beekeepers have not received training (84.07%) on the hazards of plant protection products on their beekeeping farms. However, a large amount of beekeepers (70.73%) acknowledged that the use of plant protection products could be harmful to their activity. Hives are usually installed in or near the fields. The plant protection products used for crop protection are herbicides (27%), insecticides (23%), fungicides (8%), but especially mixed (42%).

**Conclusion:**

The results show that beekeeping in Burkina Faso remains traditional and is practiced for sociocultural reasons. The use of pesticides close to beekeeping could play a role in bee colony collapse taking place in these regions. Training beekeepers on the dangers of the chemicals they use in fields near hives is therefore essential.

## Background

Beekeeping is the breeding of bees for the purpose of producing mainly honey, then secondarily pollen, propolis and royal jelly [1, 2]. Bees have been domesticated by humans for a very long time because they reproduce under conditions set by humans, particularly in the context of beekeeping [3]. The honeybee is an arthropod belonging to the class Insects, the order Hymenoptera, the family Apidae, the genus *Apis* and the species *Apis mellifera* Linnaeus (1758). This species limits its foraging field to a few plant species from which it derives its main resources, namely nectar, pollen and resin [4]. The main subspecies found in Burkina Faso is *Apis mellifera adansonii* Latreille (1804).

Nowadays, there is a growing interest in honeybees worldwide, probably because of their food, economic and environmental importance [5, 6]. Beehive products are natural that are valued around the world for their many food, nutritional and medicinal benefits [7, 8]. The exploitation and marketing of beehive products allows practitioners to diversify their source of income and contribute to the improvement of their living conditions [9, 10]. Environmentally, beekeeping maintains a high population of pollinators useful for vegetation, horticulture and agriculture [11–13]. Honeybees therefore play an important role in the conservation of biodiversity in general and phyto-diversity in particular [14–16].

Beekeeping in Burkina Faso, and broadly in Africa, has been practiced for centuries [17]. Honey production is thus an important activity in the rural economy [9, 18, 19]. In Burkina Faso, many international structures (FAO, EU, etc.) support beekeepers in improving their beekeeping activity [20]. In spite of these efforts, beekeeping is still a secondary and complementary activity to agriculture, the main activity of the primary sector [21].

In order to improve their agricultural production, farmers carry out phytosanitary treatments based on synthetic chemicals to control pests and diseases that can cause enormous damage to crops [22]. Due to the expansion of cropping areas observed in recent decades **in **Burkina Faso [22, 23], a few areas are exploited for beekeeping [20]. Beekeepers who are mostly farmers store their hives in or near their fields. Worker bees through their activities can come into contact with these products, with considerable consequences. In developed countries, much work on the effects of agricultural practices and agrochemicals on bees has been carried out [24–28]. These work has highlighted the negative consequences that these can have on honeybees. These consequences are physiological and behavioral [29–32] impact bees and can therefore weaken a colony by causing mortalities of individuals. However, in most developing countries in general, and more particularly in Burkina Faso, studies of colony collapse are scarce [20, 33]. Studies have been focused on the identification of melliferous plants [17, 34–38] and knowledge of the entomofauna cohabiting in the hive with honey bees in Garango which is in the Eastern part of Burkina Faso [39]. Specific studies on the interaction between agricultural practices and honeybee colonies have not yet been concretely carried out. Given the importance of bees in pollinating plants and maintaining biodiversity [40–42], knowledge of this interaction is essential.

The objective of this study is to characterize beekeeping operations and to evaluate, through a survey, the level of knowledge of beekeepers on the impact of the use of pesticides on honey bee colonies. Specifically, it aims to evaluate the use of pesticides in honey production areas, to assess beekeepers' knowledge on the interaction between agricultural-beekeeping practices and to examine the difficulties encountered with regard to pesticide treatments.

## Methods

### Study sites

**Fig. 1:**
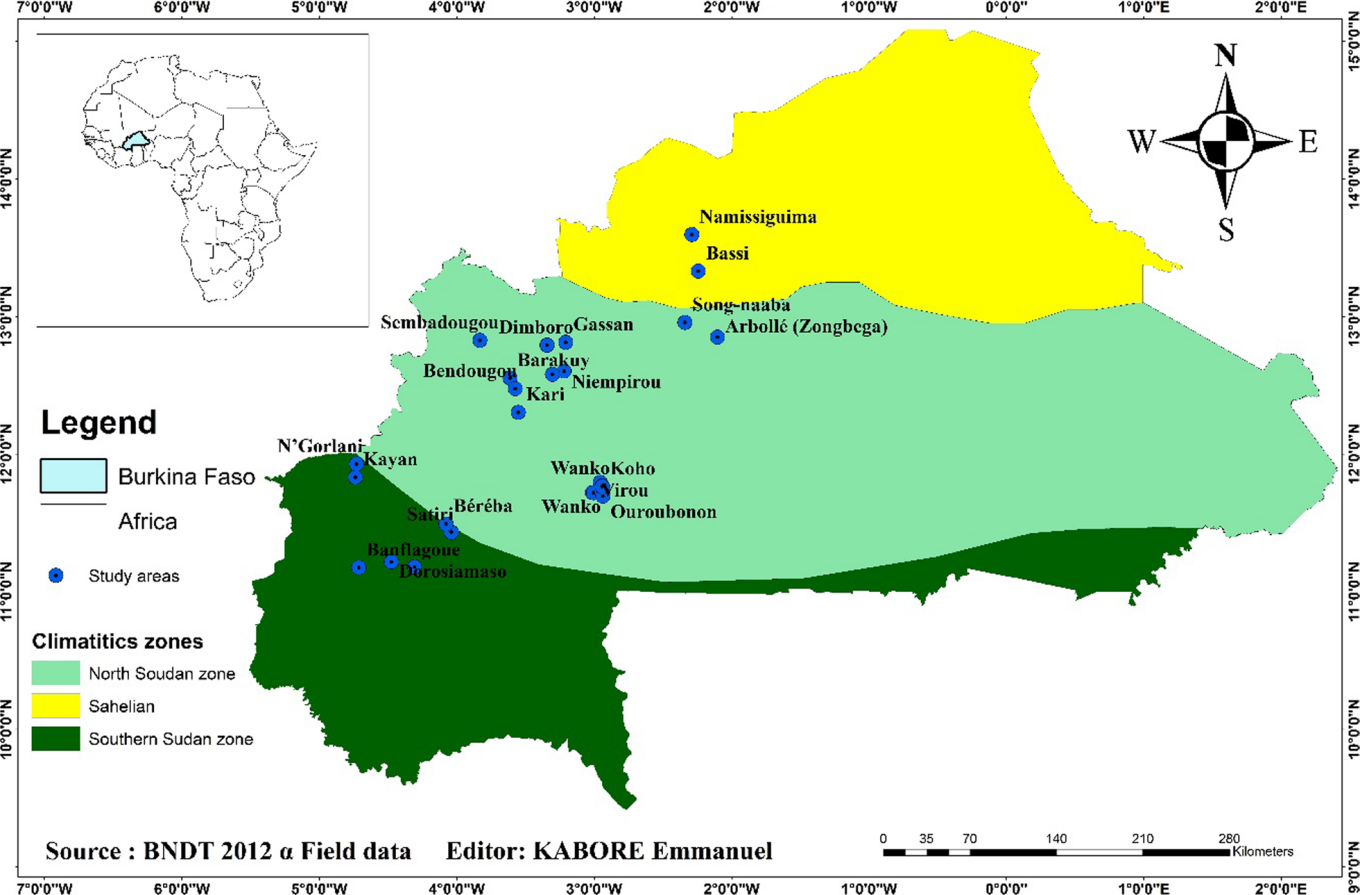
Mapping of study sites

This study was carried out in three (03) administrative regions located in the western and northern zones of Burkina Faso (Fig. 1). These regions are Boucle du Mouhoun, Hauts-Bassins and Nord. This choice is justified on the one hand by the importance of agricultural production associated with the massive use of phytosanitary products [22] and on the other hand by the significant production of honey in these regions of Burkina Faso [20]. Each of the study regions is under the influence of different agro-climatic zones. Hauts-Bassins region is located in the South Sudanian phytogeographical zone, while Boucle du Mouhoun and Northern regions are located in the North Sudanian and Sahelian phytogeographical zones, respectively [43].

### Data collection and processing

A survey was conducted in 22 randomly selected villages in the three regions. The choice of farmers to be investigated was made randomly among those who engaged in both agricultural and beekeeping activities. It consisted of administering 113 people through direct interviews, a questionnaire previously made. The questionnaire submitted to beekeepers included questions relating to socio-demographic information, beekeeping operations, phytosanitary treatments carried out in the vicinity of the apiary and the perception that beekeepers have of the dangers of pesticides on their bee colonies. An exploratory survey of eight farmer beekeepers per region allowed us to determine the size of the sample we surveyed (113 peoples), using the normal approximation of the binomial distribution proposed by [44]:

$${\mathbf{N}} \, = \, {{\varvec{\upmu}}}^{{\mathbf{2}}}_{{{\mathbf{1}} - \, {{\varvec{\upalpha}}} \, /{\mathbf{2}}}} \frac{{{\mathbf{Pi}} \left( {1 - {\mathbf{Pi}}} \right)}}{{{\varvec{\updelta}}}}$$

where N is the sample size (number of farmer beekeepers to be surveyed in the three study areas). μ1-α/2 represents the value of the normal random variable for a risk α 0.05. µ1-α/2 = 1,96.

Pi represents the proportion of farmer beekeepers who are aware of the adverse effect of plant protection products on bee colonies.

The δ margin of error for any parameter to be estimated from the survey is 5%.

The number of people to be investigated by region (72 in the Boucle du Mouhoun, 25 in the Hauts-Bassins and 16 in the North) was determined by proportionality by considering the number of beekeepers in each region [5, 20].

### Data analysis

The data obtained were entered and coded on an Excel 2016 spreadsheet, which was also used to determine descriptive statistics (percentage and average), as well as to draw graphs. Data on education level, deposition of hives in crop fields, use of plant protection products and behavior were subject to multiple correspondence analysis (MCA) using R software version R-4.3.1.

## Results

### Socio-demographic characteristics of farmer beekeepers in the study area

An analysis of the results recorded in Table 1 shows that in these three regions covered by the survey, beekeeping is a secondary activity (96.47% of respondents) mainly carried out by men (90.27% of respondents). The majority of beekeepers surveyed are married (84.96%). 40.7% of the interviewees are illiterate and 20.42% are literate; 15.04% and 12.38% reached the primary and secondary levels, respectively. Only 15.93% of stakeholders have received at least one training on the dangers of using plant protection products on honey bee colonies (Table 1). More than half of the respondents (58.40%) inherited the activity from their parents. Young beekeepers (age < 30 years) represent only 7.97% of all respondents, compared with 92.03% who are adults (30 to 72 years) (Table 1).

**Table 1:** Socio-demographic characteristics of the peasant beekeepers surveyed

Characteristics	Percentage value (%)
*Gender*	
Man	90.27
Woman	9.73
*Marital status*	
Married	84.96
Single	15.04
Divorced	00
*Place of the activity*	
Main activity	3.53
Secondary activity	96.47
*Source of motivation for the activity*	
Parental heritage	58.40
Personal initiative	32.76
Intermediate of a structure	8.84
*Age range (years)*	
[20–29]	7.97
[30–39]	21.24
[10, 40–48]	28.32
[49–58]	30.97
[60–69]	10.61
[70–79]	0.89
*Neither level*	
No level	40.7
Alphabetized	20.42
Koranic school	9.73
Primary	15.04
Secondary	12.38
Higher	1.73
*Training (s) on the dangers of using plant protection products on honeybee colonies*	
Yes	15.93
No	84.07

Table 2 shows that the largest number of respondents (40.79%) have been engaged in beekeeping for no more than nine years.

**Table 2:** Distribution of farmer beekeepers according to their seniority in beekeeping activity

Duration (year (s))	[0–9]	[10–19]	[20–29]	[30–39]	[10, 40–48]	Total
Number of people	54	29	21	7	2	113
Frequency (%)	47.79	25.67	18.58	6.19	1.77	100

### Characteristics of beekeeping in the regions of Boucle du Mouhoun, Hauts-Bassins and Nord

#### Average number and typology of beekeepers' hives

##### Average number of beekeepers’ hives

The 113 farmer beekeepers surveyed have a total of 2489 hives, an average of 22.02 ± 15.04 hives per respondent. Among the hives, 791 are colonized or 31.77%. The majority of respondents, 61.07%, own one to 20 hives. Twenty-three point eighty-nine percent of respondents (23.89%) have a number of hives between 21 and 40, while for 15.04% this number is greater than 40.

##### Typology of beekeepers’ hives

**Fig. 2:**
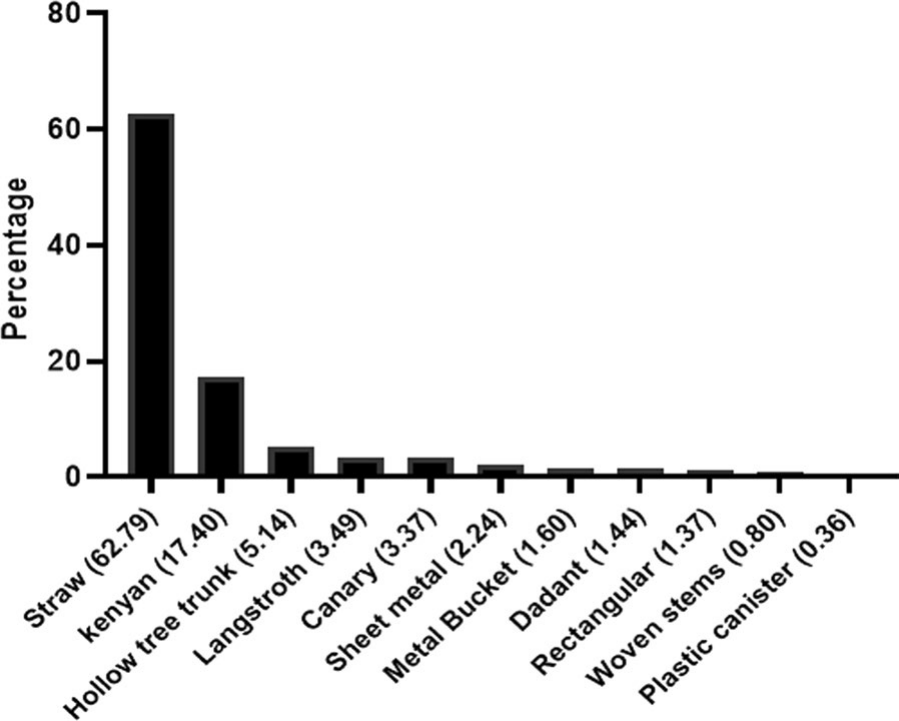
Typology of hives identified in the three regions during the survey

**Fig. 3:**
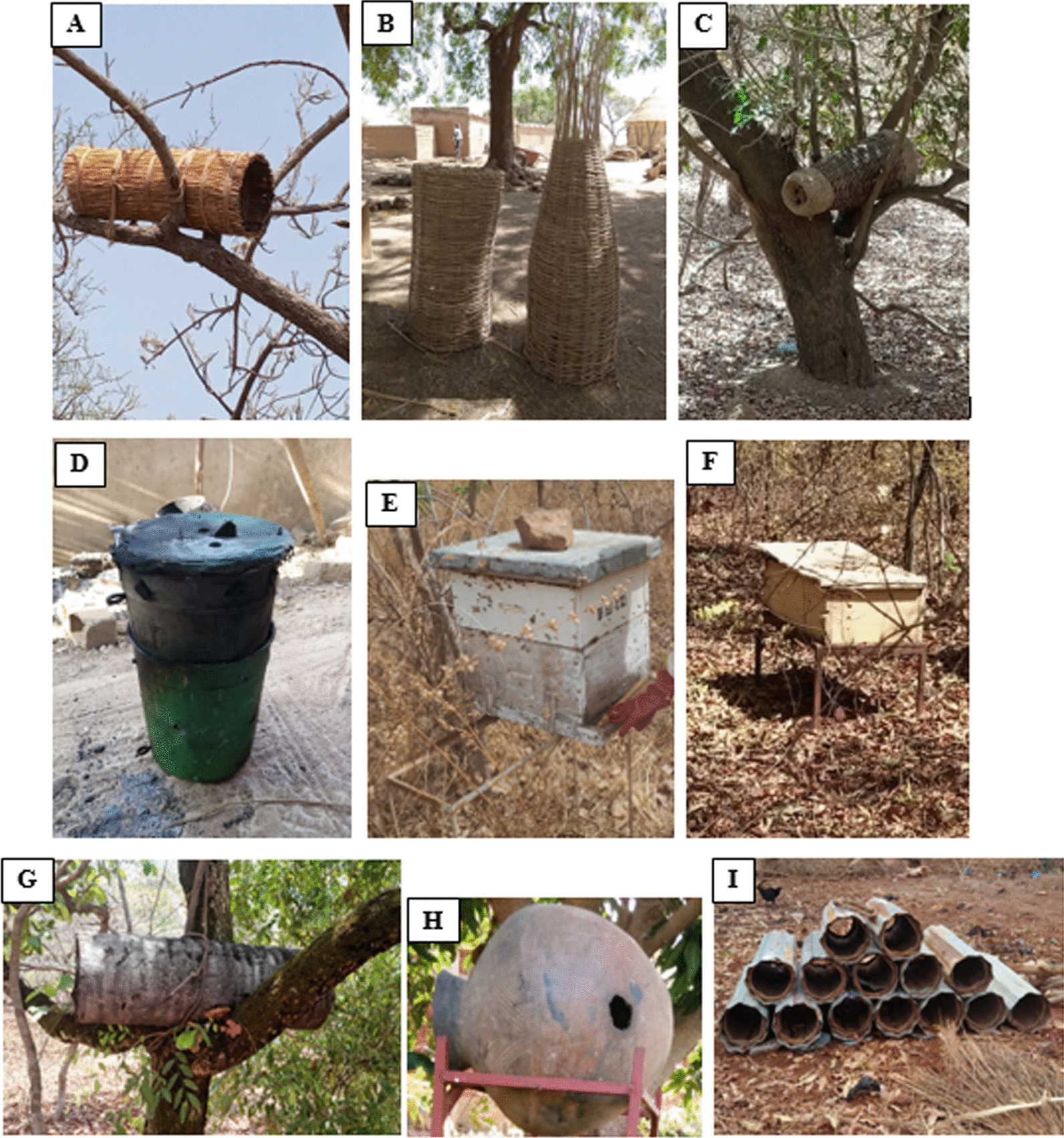
**A** Straw hives installed in a tree.** B** Hives made of woven stems (*Combretum micranthum*) in confection.** C** Hives made of woven stems installed in a tree (*Andropogon gayanus*).** D** Hive in metal Bucket.** E** Dadant hive.** F** Rectangular hive.** G** Hollow tree trunk hive. **H** Canary hive **I** Sheet metal hive

According to the respondents, traditional straw hives (used by 62.79% of them) and modern Kenyan-type hives (used by 17.4% of them) are the two types of hives that are mainly used in these regions (Fig. 2) although other types of hives made using local knowledge have been identified (Fig. 3).

#### Harvesting of honey and other bee husbandry products

**Fig. 4:**
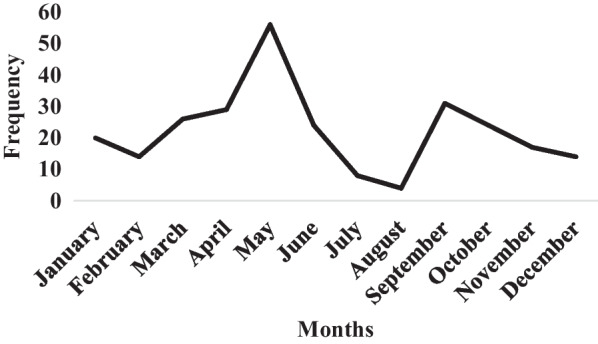
Periodicity of annual honey harvests by respondents

The timing and number of honey harvests vary from beekeeper to beekeeper. However, the majority of beekeepers, or 55.75% of respondents, harvest their honey two (02) times a year. The analysis of Fig. 4 shows that these harvests are made according to the periods of large honey flows (April–May) and small honey flows (September–October). Four products of bee farming are exploited by the respondents. This is honey exploited by 100% of the beekeepers surveyed; wax, pollen and propolis exploited, respectively, by 30.08; 16.81%; and 4.16% of beekeepers surveyed (Table 3).

**Table 3:** Technical characteristics of beekeeping in the regions surveyed

Harvested products			
Denominations			Frequency (%)
Honey			100
Wax			30,08
Pollen			16,81
Propolis			4,16

Techniques and types of bait used for harvesting			
Techniques used	Bait types		Frequency (%)
*Smoking*	Cow dung		15,04
	Plant parts	*Vitelleria paradoxa* Gaertn	22,12
		*Eucalyptus camaldulensis* Dehn	6,19
		*Sclerocaria birrea* (A. Rich.) Hochst	5,3
		*Diospyros mespiliformis* Hochst. ex A. Rich	4,42
		*Parkia biglobosa* (Jacq.) R.Br. ex Benth	8,85
		*Combretum* sp.	4,42
		*Lannea microcarpa* Engl. & K. Krause	1,8
		*Khaya senegalensis* (Desv.) A. Juss	2,65
		*Guiera senegalensis* J. F. Gmel	5,33
		*Piliostigma* sp.	7,96
		*Mangifera indica* L	3,53
*Daub*	Wax		12,39

In order to bait bees, beekeepers mainly use the smoking technique using parts of the plants (leaves, bark, fruits and roots) and/or cow dung. Only 12.39% of these beekeepers use the technique of brushing the inside of their hive(s) using melted wax (Table 3).

### Perception of the consequences of the use of agrochemicals by respondents in the study areas

#### Main speculations produced by respondents and associated plant protection products

##### Main speculations produced by respondents

**Fig. 5:**
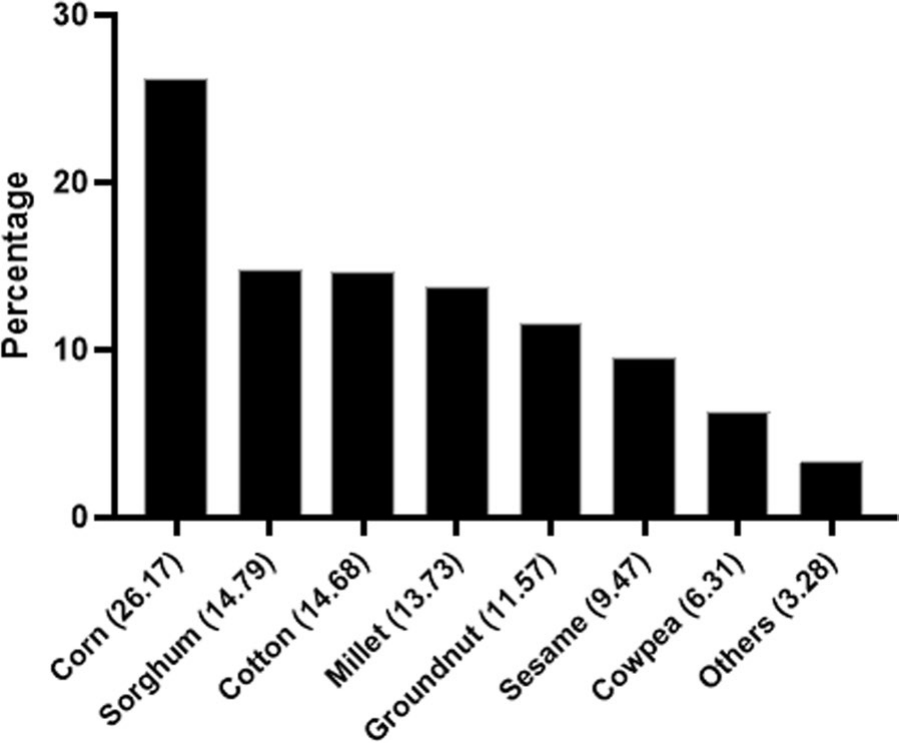
Main crops identified among respondents in the study area

Corn (*Zea mays* L.) (grown by 26% of respondents), sorghum (*Sorghum bicolor* L. Moench) (grown by 15% of respondents) and cotton (*Gossypium hirsutum* L.) (cultivated by 15% of respondents) are the three main crops sown by the surveyed population. Millet (*Pennisetum glaucum* L.), groundnut (*Arachis hypogaea* L.), sesame (*Sesamum indicum* L.) and cowpea (*Vigna unguiculata* L. Walp.) are also widely cultivated by this population (Fig. 5).

##### Main plant protection products used by respondents to control pests

**Fig. 6:**
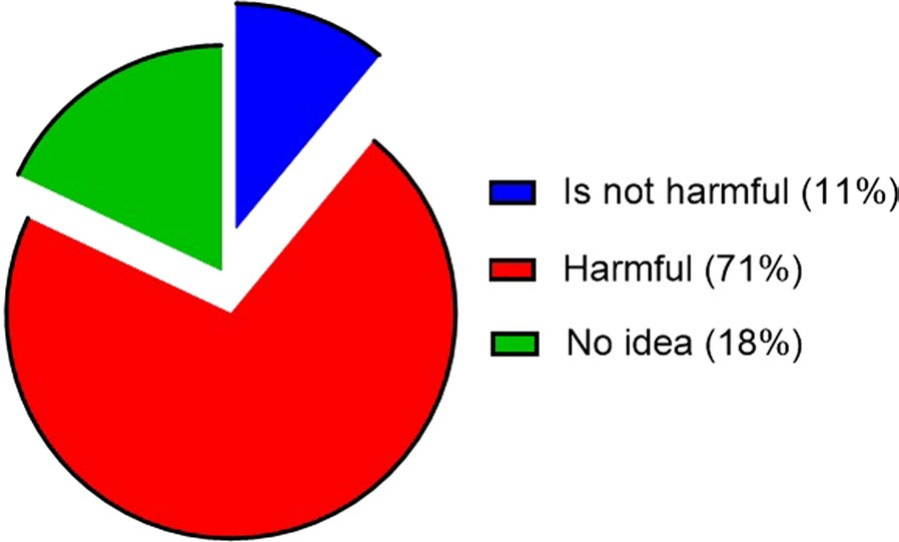
Perception of farmer beekeepers on the consequences of the use of plant protection products

Three types of plant protection products are applied by respondents to crops near hives. Insecticides, fungicides and herbicides are applied by 23%, 8% and 27% of respondents, respectively. 42% of respondents use these three plant protection products in combination. The analysis of Fig. 6 shows that the majority of respondents (71%) are aware of the harmful effects of plant protection products on bees. On the other hand, 18% of respondents say they are unaware of these effects.

#### Correlation between the use of plant protection products and the difficulties encountered in beekeeping

**Fig. 7:**
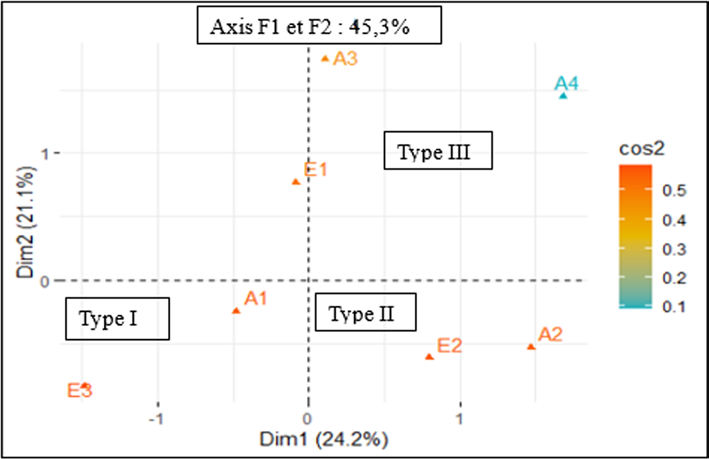
Relationship between respondents' education levels and the location of hives in their fields. A = Education, A1 = Illiterate, A2 = Primary, A3 = Secondary, A4 = Higher E = Location of hives in their fields; E1 = Low location (0-10%), E2 = Medium location (11-50%), E3 = High location (51-100%)

With regard to the relationship between the level of education and the location of hives in their fields, the first two axes F1 and F2 explain 45.3% of the overall variability (Fig. 7). The first axis F1 contributes to 24.23% and the second axis F2 to 21.11%. There is therefore a correlation between the level of education and the percentage of location of hives in crop fields. The results indicate that those who are illiterate tend to keep the maximum of their hives in their crop fields (Type I). In addition, those with a higher level of education tend to move their hives as far away from their cultivable area as possible to prevent phytosanitary treatment products from causing damage to their bee colonies (Type II and Type III).

**Fig. 8:**
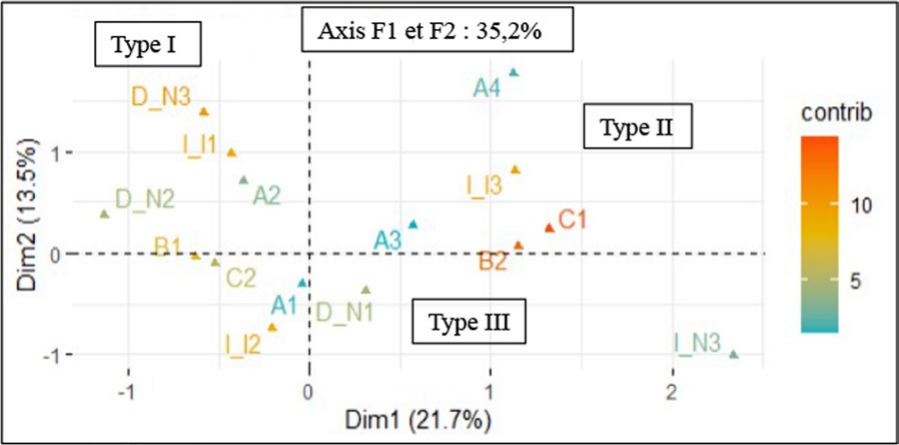
Relationship between the use of plant protection products and the behavior of bees. I: Pesticide dosage, I1: Low dosage, I2: Medium dosage, I3: Overdose; N1: Harmful, N2: Not harmful, N3: No idea; B: Bee behavior B1: Normal, B2: Abnormal C: Fall or no bees; C1: Fall, C2: No fall

From the point of view of honey bee behavior in relation to phytosanitary treatments carried out, the first axis F1 contributes to 30.29% and the second axis F2 to 18.44% (Fig. 8). The two axes therefore explain 48.73% of the overall variability. These results show that there is a correlation between the fall of bees, the abnormal behavior observed in bees and the increased use of phytosanitary products by beekeepers. The use of pesticides in overdose would be a cause of abnormal behavior and bee falls noted by some practitioners.

## Discussion

This study allowed us to better understand the beekeeping practices **used** by farmers beekeepers in the Boucle du Mouhoun, Hauts-Bassins and Northern regions of Burkina Faso. The results showed that beekeeping is mostly practiced in these regions by men who inherited it from their parents. As a secondary activity, beekeeping is compatible with other occupations and can thus contribute to the creation of jobs and additional income [7, 45]. In general, in Burkina Faso we note the predominance of men in the activities of the primary sector and this could be explained by local sociocultural realities, making them heads of families in search of financial resources for family needs [46]. This predominance of men in beekeeping activity in Burkina Faso had already been reported during the census of beekeepers by [20]. However, the intervention of several programs and projects promoting the gender through the provision of beekeeping equipment and financial resources has made it possible to identify a few female beekeepers among those interviewed. The majority of beekeepers surveyed were married, which leads us to hypothesize that beekeeping is a source of income diversification for them [10, 47]**.** Young people under the age of 30 are less involved in beekeeping. This observation has been made by two other authors in the subregion, namely Yédomonhan [48] in Benin and Koudegnan et al. [49] in Togo. Indeed, these able-bodied people do not clearly perceive the profit that beekeeping can bring them, and so prefer to focus on activities with immediate income. Also, according to [20], there are mystical considerations surrounding the activity that make it a senior citizen affair. This could partly explain these results.

There are several reasons for the low proportion of women in the survey population. Firstly, their lack of technical skills in the search for raw materials and their lack of knowledge of hive-making techniques do not give them an advantage over men. Secondly, one of the main reasons why many women abstain from this activity is linked to the aggressiveness of *Apis mellifera adansonii* bees [50]. *Apis mellifera adansonii* is very aggressive, and this may be linked to genetic and environmental factors (difficult weather conditions at certain times of the year, availability of honey resources, etc.) [50]. However, with the promotion of modern beekeeping, awareness-raising work and the socio-anthropological deconstruction of mystical beliefs should make it possible to interest young people and women in beekeeping. The use of modern hives could also be an important alternative to women's participation in beekeeping activities, as these hives are easy to handle.

In general, in the regions surveyed, apiaries are mostly made up of traditional hives made from local materials and crafted using local knowledge. These types of hives are well known throughout West Africa [20, 51, 52]. This predominance of this type of hive could be explained on the one hand by the local availability of materials (straw, clay, stems, tree trunk, etc.) essential to their design and on the other hand, by the know-how of beekeepers which is transmitted in a generational way [52]. Modern hives, which are less widely used due to their high cost, are considered inaccessible and require training and modern equipment (harvesting gear, smoker, frame lifter, bee brush, etc.), which are also expensive. However, they are appreciated for their ease of operation. The high cost of modern hives and beekeeping equipment does not encourage modernization of the beekeeping sector in these regions. However, there is a slow trend toward beekeeping using modern means thanks to the constant support of financial structures (Beekeeping Centers), some NGOs and especially thanks to the awareness of some beekeepers on the advantages of modern hives (ease of monitoring and harvesting) [20]. The peasant beekeepers surveyed have an average of 22 hives. This number is relatively low compared to those recorded in other countries by other authors, including [45] in the Central African Republic (40 to 70 hives) on average per beekeeper and [53] in Ivory Coast with an average of 105 hives per farmer beekeeper interviewed. This difference could be justified by the fact that beekeeping is still considered a secondary activity in Burkina Faso [37, 54].

Hives are mainly populated by wild colonies attracted to the hives by smoke and/or wax [55]. Beekeepers explain that the smoke produced by these plant parts gives off a pleasant scent for the bees. According to them, the speed with which the hive is populated can depend on the effectiveness of the smothering. In our study, 11 plant species were identified and used by beekeepers for smothering [37] had counted 13 in two agro-ecological zones in Burkina Faso. In fact, the practices and substances used for smothering vary from one zone to another. While some claim to use these products because they are traditional, others claim to have learned to do so during training courses. Further research could lead to the synthesis of inexpensive, environmentally-friendly swarm attractants. This would avoid the direct use of plants and help reduce the cost of any imported beehives in a context of beekeeping modernization.

Honey is harvested in two main phases. The first, or honey flow, takes place between February and June. The second takes place between August and November. According to Sawadogo [50] and Nombre et al. [56], the honey flow periods coincide with the flowering peaks of ligneous and herbaceous plants, between February–June and August–November, respectively. The presence of honeydew flowers indicates the availability of nutrients (nectar and pollen) for bees, and therefore the likelihood of storing large quantities of honey in the hives.

In general, the beekeepers surveyed do not have a solid knowledge of the biology, pathologies and multifactorial causes that would justify the desertions of bee colonies often observed [53, 54, 57]. Hives are usually installed in agricultural plots due to the lack of space to establish their apiary. Due to the rainy seasons that usually start late, many beekeeper-farmers claim to apply herbicides in order to reduce weeding time and allow planting on time [21, 22].

The application of phytosanitary products during the flowering period of cultivated plants endangers the survival of worker bees and mainly causes contamination within the hive [58, 59]. This could explain some desertions encountered by beekeepers at this time of year. In general, chemical control of crop pests is carried out without any real consideration of beekeeping and the foraging hours of worker bees. According to some beekeepers, phytosanitary treatments are not carried out in the direction of the hives and cannot have consequences on bee colonies. Albouy [60] pointed out, however, that plant protection products would induce abnormal behavior in foragers.

## Conclusion

At the end of this participatory diagnosis, it appears that beekeeping is a secondary activity. Its practice is dominated by men and remains extensive even if we observe intensive beekeeping practiced by a minority working with modern equipment. The majority of craftsmen are without any level of education and use phytosanitary products in the vicinity of their hives. The beekeepers surveyed acknowledge that plant protection products are harmful to their bee colonies, but given the lack of space to establish their apiary and the pressure of weeds and crop pests, they are forced to use them. Studies on the direct effect of plant protection products on foraging bees and the presence of chemical residues in hive products are needed to confirm the real impact of the use of these products on beekeeping activity in these regions.

## Data Availability

All data are available in this paper.

## Abbreviations

FAO: Food and Agriculture Organization
EU: European Union
NGOs: Non-profit Organizations
MCA: Multiple Correspondence Analysis
